# Applications of Hydrogels in Drug Delivery for Oral and Maxillofacial Diseases

**DOI:** 10.3390/gels9020146

**Published:** 2023-02-09

**Authors:** Lijia Liu, Dan Wu, Heng Tu, Mengjiao Cao, Mengxin Li, Li Peng, Jing Yang

**Affiliations:** 1 State Key Laboratory of Oral Diseases, National Clinical Research Center for Oral Diseases, Department of Cariology and Endodontics, West China Hospital of Stomatology, Sichuan University, Chengdu 610041, China; 2 Key Laboratory of Bio-Resource and Eco-Environment of Ministry of Education, College of Life Sciences, Sichuan University, Chengdu 610065, China

**Keywords:** hydrogel, drug delivery system, mouth diseases, maxillofacial injuries

## Abstract

Oral and maxillofacial diseases have an important impact on local function, facial appearance, and general health. As a multifunctional platform, hydrogels are widely used in the biomedical field due to their excellent physicochemical properties. In recent years, a large number of studies have been conducted to adapt hydrogels to the complex oral and maxillofacial environment by modulating their pore size, swelling, degradability, stimulus-response properties, etc. Meanwhile, many studies have attempted to use hydrogels as drug delivery carriers to load drugs, cytokines, and stem cells for antibacterial, anticancer, and tissue regeneration applications in oral and maxillofacial regions. This paper reviews the application and research progress of hydrogel-based drug delivery systems in the treatment of oral and maxillofacial diseases such as caries, endodontic diseases, periodontal diseases, maxillofacial bone diseases, mucosal diseases, oral cancer, etc. The characteristics and applications of hydrogels and drug-delivery systems employed for the treatment of different diseases are discussed in order to provide a reference for further research on hydrogel drug-delivery systems in the future.

## 1. Introduction

The oral and maxillofacial region bears various critical functions of the human body, such as mastication, swallowing, speech, and appearance. Oral health is crucial to patients’ overall health. No matter what anatomical structures suffer damage, e.g., pulpitis, periodontitis, alveolar osteitis, oral mucosal disease, and oral cancer, almost all lesions will do harm to patients’ physiological and mental health, and influence their social living. Hence, preventing and treating oral diseases is of vital significance to improve patients’ quality of life.

A suitable drug-delivery system (DDS) proves a powerful therapeutic tool for the thorough and efficient treatment of oral diseases, demanding biomaterials with excellent performance as drug-delivery platforms [1]. Conventional DDS mainly consists of oral gels, tablets, and lozenges. However, not only the complexity of oral and maxillofacial structures but also the specificity of the oral environment leads to the challenges of drug delivery to the lesion region. For example, there are many fascias and cavities in the maxillofacial region, which usually result in the spreading of infection and the difficulties of infection control. Only sustaining long-term and effective drug concentration in the infectious region can control the infection well [2]. What’s more, the elevated moistness of the oral environment and the movement of the tongue makes it more troublesome to control the therapeutic patch at the lesion site consistently, which brings problems to the prevention and treatment of oral mucosal diseases [3]. As a consequence, developing an on-demand and practical DDS becomes fairly essential and urgent.

**Figure 1:**
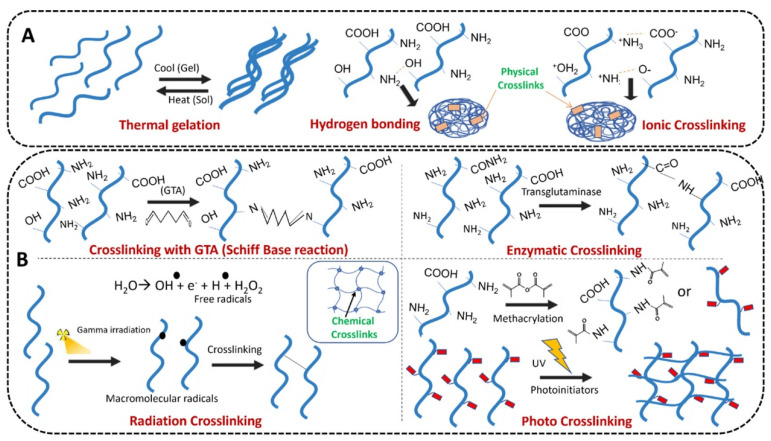
(**A**) physical and (**B**) chemical crosslinking techniques employed for gelatin hydrogel formation. Reprinted with permission from Ref. [10] Copyright 2022 Elsevier.

Hydrogels have demonstrated unique properties of their structure and performance, which discriminate them from other biomaterials [4]. Hydrogel is a biological material by physical or chemical crosslinking reactions of monomers, forming a polymer network system [5]. The specific reactions are presented in Figure 1. For instance, the hydrogel, a methacrylate-based polymer, was prepared with the combination of N-vinylpyrrolidone (NVP), 2-hydroxyethyl methacrylate (HEMA), and glycerol monomethacrylate (GMMA) monomers with methacrylic acid (MA). These monomers could be crosslinked based on the radical polymerization initiated by thermic, ultraviolet (UV), or reductive-oxidative reactions [6]. Different crosslinking methods lead to different properties of hydrogels. Generally speaking, the physicochemical properties of chemical crosslinked hydrogels are relatively more tunable than those of physical crosslinked ones [7]. The network system provides a platform for various modules and cells and shows outstanding biocompatibility and biodegradability [8]. It is worth noting that the three-dimensional network structure of hydrogel shows great similarity with the extracellular matrix (ECM), which means it can provide a comparable living environment for cells and induces proliferation and differentiation [9].

**Figure 2:**
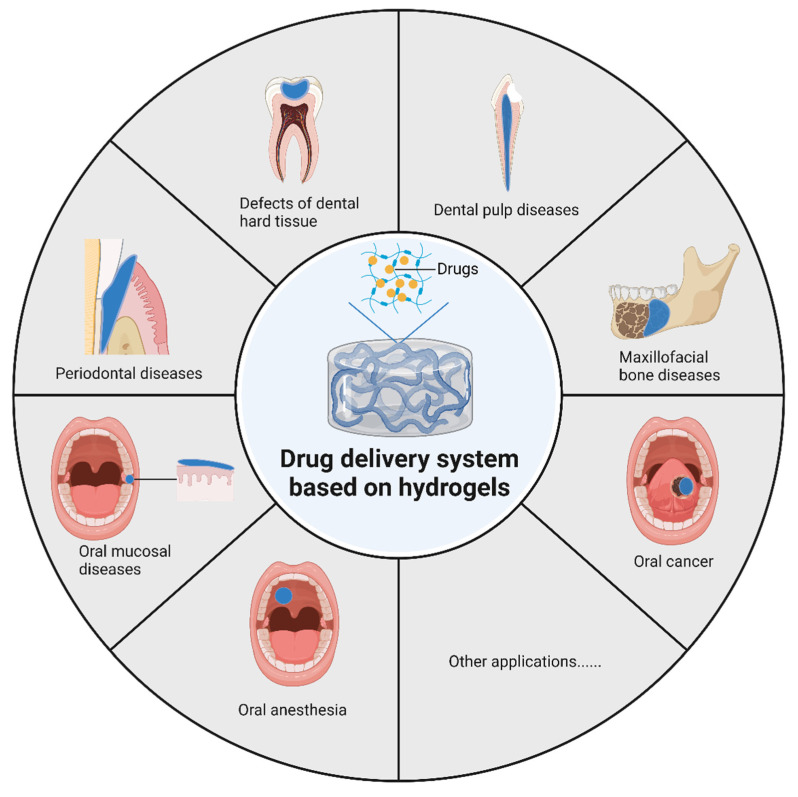
Applications of hydrogels in drug delivery for oral and maxillofacial diseases.

Above all, hydrogels have been put into application in massive fields of laboratory research and clinical treatment, covering hard and soft tissue regenerative engineering, local anesthesia, wound healing, and so on, revealing a promising prospect of DDS in the treatment of oral disease. This paper reviews the current status of hydrogel applications in oral and maxillofacial diseases, introduces the role of various hydrogels in oral drug delivery systems, and outlines future research directions. The applications of hydrogels in drug delivery for oral and maxillofacial diseases are presented in Figure 2.

## 2. Materials and Methods

In the literature search phase, the following databases were used: PubMed, Web of Science, Cochrane Library, and Embase. The literature search was first conducted using the keywords “Hydrogels”, “Drug Delivery Systems”, “Dentistry”, and their related keywords. Afterward, the search was conducted by combining different keywords for diseases with the keywords “Hydrogels” and “Drug Delivery Systems”, and the keywords for diseases used for the search included “Dental Caries”, “Dental Pulp Diseases”, “Periodontitis”, “Bisphosphonate Associated Osteonecrosis of the Jaw”, “Osteoradionecrosis”, “Mandibular Reconstructions”, “Dry Sockets”, “Peri-implant”, “Mucosal Diseases”, “Mouth Neoplasms”, “Anesthesia” and their related keywords. A total of 808 articles were found, 104 of which were approved for the writing phase, while the remaining articles were excluded due to duplication, irrelevance to drug delivery, or the absence of hydrogels in the system. Literature from 2017 to October 2022 was investigated.

## 3. Hydrogels for Oral and Maxillofacial Environment

Hydrogels, a hydrophilic polymer network, can be composed of natural or synthetic polymers. Natural polymers, such as gelatin, chitosan, sodium alginate, hyaluronic acid (HA), and so on, usually have outstanding biocompatibility and biodegradability, but their mechanical properties are often inferior. Synthetic polymers such as polycaprolactone (PCL), polyethylene glycol (PEG), Pluronic, and so on, usually have stronger mechanical properties, but often require surface modification to achieve better biological functionality [11]. Different natural or synthetic polymers can be chemically crosslinked or physically crosslinked to form hydrogels, including those with injectable, temperature-sensitive, adhesion, and other properties to achieve better local drug delivery. Chemical crosslinking connects different polymer molecules by forming covalent bonds between functional groups through certain reactions, including click chemistry, enzymatic reaction, Schiff base coupling, Diels-Alder linkage, etc., which usually provide stronger mechanical properties due to their high crosslinking density [12]. However, the toxicity of their crosslinkers needs to be considered. In contrast, physical crosslinking hydrogels are mainly gelled by non-covalent interactions such as hydrogen bonding, hydrophobic interaction, chain entanglement, van der Waals interactions, etc., which are easier to be synthesized because they do not require crosslinkers, but have relatively inferior physical properties [13].

To observe the micromorphology of hydrogels, Scanning Electronic Microscopy (SEM) inspection is used as an indispensable research tool, which requires the observation of samples in a high vacuum environment. However, the vacuum environment could not only contaminate the sample but also destroy the microstructure. Consequently, hydrogel samples are usually required to be dried before SEM inspection. The present drying means of hydrogels include freeze-drying and supercritical drying methods. The freeze-drying method is to solidify the product by freezing, and then evaporates the solvent by sublimation when heated [14]. While the supercritical drying method is to dry the hydrogel under supercritical fluid conditions, which avoids the collapse and contraction of the structure during the drying process and maintains the network framework structure of the hydrogel [15,16].

In the studies of topical drug delivery using hydrogels for oral and maxillofacial diseases, some of the studies used relatively simple hydrogels as carriers for drug delivery, either directly carrying the drug or carrying nanoparticles containing the drug to achieve continuous release of the drug. While another part of the studies further investigated hydrogel systems with different properties, such as injectability, adhesion, pH responsiveness, etc., to facilitate the topical application of hydrogel systems or to achieve controlled release of drugs under specific stimuli. The most commonly used hydrogel matrix for topical drug delivery studies in the oral and maxillofacial region is injectable hydrogels formed by chemical or physical crosslinking. Injectable hydrogels are widely used in the field of drug delivery because they are not limited by defect geometry [17] and are suitable for filling irregular root canals, periodontal pockets, bone defect areas, etc. Particularly, some injectable hydrogels exhibit a sol-gel transition in response to stimuli such as temperature, light, and pH. Among them, injectable thermosensitive hydrogels are one of the most commonly used hydrogel systems, as they usually can be injected at low temperatures and rapidly transform into gels at body temperature [18]. Thermosensitive hydrogels are temperature-sensitive materials and changes in environmental temperature can lead to changes in their sol-gel states [19]. In order to be an ideal thermosensitive hydrogel system for drug delivery, thermosensitive hydrogels should be free-flowing solutions at room temperature and transform into non-flowing gels at physiological temperatures (32–37 °C) [20]. The mussel-inspired mucoadhesive hydrogels are also often used in the field of transdermal drug delivery to the oral mucosa [21]. Compounds containing catechol structures, such as dopamine, can be used as biobased adhesives, allowing hydrogel materials to bind tightly to the oral mucosa in a moist oral environment, which contributes to the continuous targeted delivery of drugs [22]. Hydrogels with special properties for application in the oral environment are included in Table 1.

## 4. Applications in Different Oral and Maxillofacial Diseases

### 4.1. Defects of Dental Hard Tissue

Dental caries is one of the most common chronic diseases all over the world [74]. Caries can gradually destroy the hard tissues of the tooth, namely enamel, and dentin. Enamel is the hardest tissue in the human body, consisting mainly of hydroxyapatite, with 96% mineralization and high wear resistance, but it cannot be regenerated after destruction [75]. Unlike enamel, dentin consists of hydroxyapatite with a mineralization of about 70%, which can form a small amount of restorative dentin after external stimulation [76]. At present, caries lesions of enamel and dentin are usually restored clinically only by resin, silver amalgam, and other materials. The progression, cessation, or reversal of caries is related to the demineralization/remineralization process of dental hard tissue [77]. Many studies have been attempted in the remineralization of enamel and dentin using a biomimetic mineralization strategy [78]. As a drug delivery matrix with widely adjustable physical and chemical properties, hydrogels have been studied as carriers of drugs, peptides, stem cells, etc., in the restoration and remineralization of dental hard tissues.

Tooth enamel is a complex mineralized tissue [79] and the cells that make it are called ameloblasts, a type of highly specialized epithelial cell. It synthesizes and secretes amelogenin and transports calcium as well as phosphorus ions to the enamel matrix. Therefore, different studies have attempted to use calcium and phosphorus ions [80], amelogenin peptides [81], and even dental epithelial cells [32] to promote the biomimetic mineralization of tooth enamel. The positive charge of chitosan enables it to adhere to the negatively charged surface of demineralized enamel, supporting the use of chitosan for regenerating mineralized enamel tissue [81]. Therefore, a simple biomimetic mineralized hydrogel system can be prepared based on chitosan, using chitosan hydrogel loaded with inorganic ions (Ca^2+^ and PO_4_^3−^) to repair the enamel surface [80]. One study used chitosan hydrogels loaded with smaller amelogenin peptides for enamel restoration and could restore hardness to 87% of normal enamel sections [81]. A few studies have tried to add dental stem cells into the hydrogel system to promote the regeneration of dental hard tissue. Through the Schiff base reaction, Mohabatpour et al. [32] use oxidized alginate and carboxymethyl chitosan to prepare injectable hydrogel, which can be used as a cell carrier for enamel regeneration. This hydrogel can maintain the shape and vitality of dental epithelial cells within 14 days.

Similar to enamel remineralization, the remineralization, and regeneration of dentin have also attracted many scholars’ attention, including hydrogel systems with different characteristics. To design scaffolds that resemble the characteristics of natural mineralized tissue for dentin regeneration, Campodoni et al. [82] used gelatin and chitosan as a hydrogel matrix, adding synthetic mineralized flakes similar to natural mineralized tissues, which were made of gelatin and magnesium-doped-hydroxyapatite (MgHA) nanocrystals. The mineral phase MgHA is very similar to the mineral components in the natural mineralized tissues, and the mineral phase is limited to the nanometer level through the interaction with gelatin, which is conducive to biomineralization. Alaohali et al. [43] prepared an injectable hydrogel with methacrylate (MA)–hyaluronic acid (HA) and di-thiol PEG and used eosin Y as the photoinitiator, which could be gelled in situ under blue light irradiation commonly used in dentistry. Meanwhile, GSK3 inhibitor NP928 can stimulate the formation of restorative dentin. Combining NP928 with injectable hydrogel makes it appropriate for clinical dental applications.

To increase the clinical application potential of materials, some studies have explored their antimicrobial or drug-carrying ability while maintaining their restorative or remineralizing role in dentistry. Ren et al. [83] combined QP5, an amelogenin-derived peptide with a remineralization effect, with antibacterial chitosan to form a hydrogel system that not only has good antibacterial ability but also shows an excellent remineralization effect. To investigate bioactive dental resins, Takeda et al. [84] tried to improve the biocompatibility of dental resins and developed a resin that releases growth factors to promote tissue regeneration. 2-hydroxyethyl methacrylate (HEMA) and the cross-linking monomer trimethylolpropane trimethacrylate (TMPT) were manufactured into a non-biodegradable hydrogel, and the loading and release characteristics of bovine serum albumin (BSA) and fiber last growth factor-2 (FGF-2) in hydrogels were studied.

Up to now, a large number of attempts have been made to remineralize and regenerate dental hard tissue using hydrogel-driven mineralization, protein/peptide-induced mineralization, and other methods. However, it is still incapable of fully restoring the complex structure of the natural dental hard tissues [78].

### 4.2. Dental Pulp Diseases

Deep caries, crown fracture, tooth dislocation, and other etiologies may lead to injury and inflammation of the dental pulp. In such cases, endodontic treatment becomes an important treatment option to preserve the affected teeth. Based on an accurate diagnosis of the pulp condition, root canal therapy, partial pulpotomy or pulp capping can be used as treatment options [85]. In recent years, pulp regeneration has also been applied in many studies in order to further preserve and reconstruct a vital pulp [86]. Since hydrogels with different compositions have widely adaptable properties, especially those with injectable properties that are suitable to act in the narrow space inside the root canal, many studies have chosen hydrogels as carriers for intracanal drug delivery and explored their applications in pulp capping, root canal disinfection, and pulp regeneration [87,88].

#### 4.2.1. Pulp Capping

When the pulp is exposed due to caries or trauma, the exposed pulp can be covered by pulp capping agents to enhance tertiary dentin deposition, and researchers are also exploring how to achieve tooth regeneration using pulp capping agents [89]. Historically, calcium hydroxide has been the gold standard drug for pulp capping, but some studies have made other attempts at the choice of pulp capping materials [90]. In the study by Komabayashi et al. [44] on direct capping materials, calcium hydroxide was loaded into the light-cured PEGMC hydrogel and controlled calcium ion release was achieved, but the hydrogel had a cytotoxicity similar to that of the adhesive system. Some studies have explored hydrogel systems with better biocompatibility. Osmond et al. [33] used an injectable hydrogel of carboxymethyl-chitosan and diglycidyl ether with calcium phosphate nanoparticles to investigate its role in protecting exposed dental pulp. The results demonstrated that the hydrogel system is biocompatible and has a beneficial effect in inducing the proliferation and differentiation of residual dental pulp stem cells. In addition to calcium ions, growth factors were also added to the hydrogel capping material. Wu et al. [51] loaded vascular endothelial growth factor (VEGF) in chitosan/beta-glycerophosphate (CS/β-GP) to achieve continuous release of VEGF, promote odontogenic differentiation of dental pulp stem cells (DPSCs), and to explore its possible usefulness in pulp capping after pulpotomy.

#### 4.2.2. Root Canal Disinfection

Root canal disinfection is one of the key factors in the success of endodontic treatment. To ensure decontamination of the infected root canal, mechanical preparation of the root canal is usually supplemented by chemical disinfection [91]. In some studies, antibacterial agents were added to the hydrogel system to achieve a good root canal disinfection effect. Chlorhexidine (CHX), an antibacterial drug, was added to the photocrosslinked gelatin methacrylate (GelMA) hydrogel, which showed a significant antibacterial effect and could maintain a high vitality of dental pulp stem cells. That is conducive to root canal disinfection in the treatment of pulp regeneration [39]. Haseeb et al. [92] encapsulated CHX in poly(ethylene glycol)-block-poly(l-lactide) (PEG-b-PLA) nanoparticles and loaded the nanoparticles in a hydroxyethyl cellulose hydrogel for root canal disinfection. Retarded release of CHX was observed over 21 days. Many antibiotics can be used for root canal disinfection [93] in addition to the clinically used CHX. For example, Bekhouche et al. [23] encapsulated clindamycin in polylactic acid (PLA) nanoparticles to improve the antibacterial properties of injectable fibrin hydrogels and similarly obtained hydrogel systems with superior antibacterial effect and biocompatibility. Likewise, Ribeiro et al. [94] loaded clindamycin or metronidazole on fibrous particles and used injectable GelMA hydrogel for ablation of endodontic infections, showing good results and low cytotoxicity.

#### 4.2.3. Pulp Regeneration

The treatment of necrotic immature permanent teeth with open apical foramen is one of the challenges that dentists face in clinical work. Since the beginning of this century, a large number of clinical studies have reported the clinical application of pulp revascularization, which not only observed the healing of periapical lesions after surgery, but also observed the continuous development of the root, and even the restoration of pulp vitality [95,96,97].

Currently, different studies have attempted to achieve pulp tissue regeneration by two main methods: cell homing or cell transplantation [98]. The cell homing method, also known as a cell-free method, involves the use of biological signaling molecules to promote the migration, proliferation, and differentiation of endogenous stem cells [99]. In order to enable growth factors to work better in narrow root canals, many studies have chosen hydrogels with different properties to carry specific growth factors, allowing the growth factors to act continuously in the root canal. Carvalho et al. [100] used the cell homing strategy to load the secretion from stem cells of deciduous teeth into the chitosan hydrogel, so as to provide continuous and controlled release of several trophic factors. Similarly, some studies attempted to incorporate drugs to promote cell differentiation, migration, and regeneration. Soares et al. [101] incorporated simvastatin into chitosan hydrogels to achieve a controlled release of drugs and increase the chemotaxis and regenerative capacity of human dental pulp stem cells (hDPSCs). Another drug, iloprost, has also been added to the injectable thermosensitive hydrogel PF127 for inducing pulp regeneration due to its ability to increase the expression of VEGF [46].

**Figure 3:**
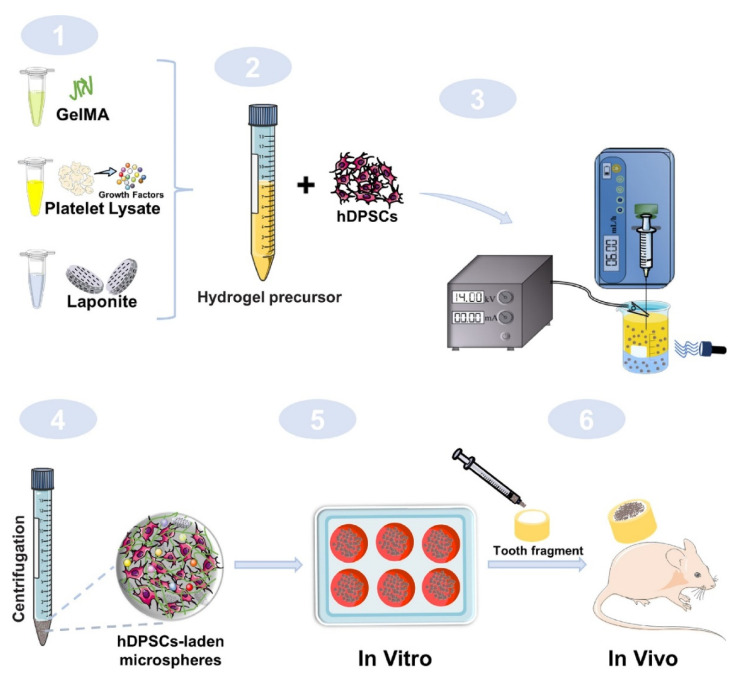
Schematic diagram of fabrication of GelMA/PL/Laponite microspheres and its application for endodontic regeneration. (1) GelMA, Platelet Lysate, and Laponite formed a hydrogel precursor. (2) Hydrogel precursors mixed with hDPSCs to make microspheres. (3) Preparation of microspheres by electrostatic microdroplet method. (4) Microspheres were collected after centrifugation and observed under the microscope. (5) In vitro biological experiments including the viability, spreading, proliferation, and differentiation behaviors of hDPSCs encapsulated in the microspheres, as well as the effects of released PL-derived GFs on tube-formation of HUVECs and hDPSCs migration behavior were systematically carried out. (6) In vivo studies were done to further evaluate whether this hybrid microsphere system could facilitate angiogenesis and histogenesis when subcutaneously implanted in immune deficiency mouse model. Reprinted with permission from Ref. [40] Copyright 2021 Elsevier.

The cell transplantation method mainly uses stem cells from a host or allogeneic source for isolation and in vitro expansion, implanted in a scaffold, and transplanted into the root canal for regeneration [40,102]. Growth factors also play an important role in cell transplantation, so many studies have incorporated growth factors in hydrogel scaffolds to promote the growth and differentiation of transplanted hDPSCs. Zhang et al. [40] used GelMA to fabricate a microsphere for the delivery of pro-angiogenic growth factors and improved the drug delivery ability of the microsphere by modifying it with the nanoclay Laponite (Figure 3). Silva et al. [24] added platelet lysate (PL), which releases platelet-derived growth factor (PDGF) and VEGF, to injectable HA hydrogels, and the stability of the material was enhanced by fibrin nanocrystals. This injectable hydrogel could not only continuously release growth factors to promote stem cell recruitment and vascular growth, but also exhibit support for the growth of hDPSCs. Park et al. [41] worked on promoting the differentiation of hDPSCs. They coupled BMP-mimetic peptide into GelMA hydrogels and used 3D printing to fabricate the material, which promoted the odontogenic differentiation of hDPSCs. In practical applications, the effects of the hydrogel system on the adhesion, chemotaxis, proliferation, and differentiation of pulp cells are all very important. Anitua et al. [103] investigated the effects of adding either hydroxyapatite or plasma rich in growth factor (PRGF) to gelatin and alginate on human pulp cells. Cell adhesion and chemotaxis were significantly increased when PRGF was added, while the combination of hydroxyapatite and PRGF promoted cell proliferation and stimulated osteogenic differentiation.

Up to now, although many studies have shown that the addition of different biologic factors or drugs in hydrogels promotes the proliferation and differentiation of DPSCs, there are still no studies that have achieved real pulp regeneration. Pulp regeneration still has a long way to go.

### 4.3. Periodontal Disease

Periodontitis is an inflammatory process that occurs in the tissues surrounding the teeth due to plaque accumulation on the teeth [104] and is mainly associated with anaerobic bacteria such as *Porphyromonas gingivalis* and *Treponema denticola* [105]. Periodontitis is treated by mechanical scaling, combined with topical antibacterial medication and occasional systemic antibacterial therapy [106]. Bacterial control in periodontal pockets and regeneration of alveolar bone are two important aspects of periodontal treatment. Thus, drugs with anti-inflammatory, antibacterial, or osteogenic effects can be selected for the topical treatment of periodontitis [107,108].

As a highly hydrated polymer network, hydrogels are commonly used in periodontal tissue formation and drug delivery [109]. For convenient administration in periodontal pockets, injectable hydrogels are widely used for drug delivery in periodontal disease [31,34,62,110]. Usually, loading drugs into hydrogel systems is designed to prolong the action or release of the drug. For example, Wang et al. [36] incorporated minocycline into injectable in situ hydrogels composed of poly(lactide-co-glycolide) (PLGA) and N-methylpyrrolidone (NMP) for periodontal antibacterial. Minocycline sustained release for more than 48 h after initial rapid release. In addition to the sustained release of drugs, the controlled release of drugs can also be achieved by special injectable drug delivery systems. Wang et al. [2,38] loaded doxycycline and lipoxin into PLGA microspheres and dispersed them in polyisocyanopeptide (PIC) hydrogels. The hydrogel system is injectable and structurally stable, and the release rate of the drug can be adjusted by adjusting the loading ratio of acid-terminated and ester-capped PLGA microspheres. For the treatment of chronic periodontitis combined with diabetes mellitus, Zhao et al. [35] developed a drug delivery system for ROS-triggered drug release in which oxidized dextran and phenylboronic acid-functionalized poly (ethylene imine) form an injectable hydrogel via a Schiff-base reaction, and the anti-inflammatory drug doxycycline and the osteogenic drug metformin were bound in the hydrogel network through B−N coordination. The release of the drug is triggered by excessive ROS due to hyperglycemia to achieve synergistic therapeutic effects of anti-inflammatory, antibacterial, and osteogenic effects.

**Figure 4:**
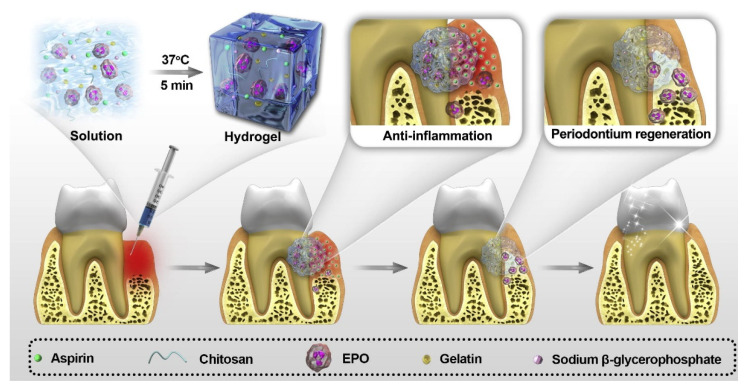
Schematic illustration of the preparation and application of the CS/β-GP/gelatin hydrogels. Reprinted with permission from Ref. [112] Copyright 2019 Elsevier.

Based on injectable hydrogels, some studies have used injectable thermosensitive hydrogels, which are able to form hydrogels in situ at body temperature after injection into the periodontal pocket, making the administration of the hydrogel in the pocket more convenient and effective [49,54,111]. Hydrogels prepared with CS and β-GP are one of the most commonly used injectable thermosensitive hydrogels for the treatment of periodontitis. Zang et al. [53] added ornidazole and BMP-7 simultaneously to hydrogels formed from CS and β-GP for the treatment of bone loss and inflammation that typically exist together in patients with severe periodontitis. The results showed that the hydrogel system could release the drug stably and consistently. Some studies introduced gelatin into the crosslinking of CS and β-GP to reduce the gelation time by electrostatic interactions [112]. Xu et al. [112] prepared a thermosensitive hydrogel for the sustained release of aspirin and erythropoietin (EPO) using CS, β-GP, and gelatin, and drug release could last for at least 21 days (Figure 4). Poloxamer 407 (Pluronic F127) is also one of the most commonly used thermosensitive polymers. Chen et al. [48] used injectable thermosensitive hydrogel based on Pluronic F127 to deliver simvastatin locally to treat periodontitis and reduce periodontal bone loss. On the contrary, a study used a hydrogel system that maintains the gel shape at low temperatures and transforms it into liquid at body temperature [55]. They added BMP-6 to an injectable thermosensitive chitosan/gelatin/glycerol phosphate hydrogel, which was able to reduce inflammatory progression and promote periodontal regeneration.

Inflammation leads to a decrease in pH, and a slightly acidic environment of pH 5.0 to 7.0 is conducive to the growth of many periodontal pathogenic bacteria [113]. When pH decreases, the pH-responsive hydrogel drug release system is able to achieve natural control of inflammation [114]. Based on the pH changes at the inflammation site, some studies have attempted to modulate the local drug release by pH for on-demand and controlled drug delivery, especially in the slightly acidic inflammatory environment. Bako et al. [45,115] made a visible light polymerized hydrogel for the co-release of metronidazole and CHX, which allows rapid gel formation under blue light commonly used in dentistry. The methacryloil-group modified polymer and methacrylated-poly-γ-glutamic acid nanoparticles can work together as a pH-sensitive drug delivery system, with lower pH resulting in greater drug release. Chang et al. [113] incorporated naringin into a pH-responsive hydrogel with thermogelling properties. This hydrogel system composed of carboxymethyl hexanoyl chitosan (CHC) and β-GP was able to significantly reduce periodontal bone loss, and the release of naringin is faster at lower pH conditions. In addition, lower pH can also regulate drug delivery by modulating the drug carrier structure. Li et al. [116] attempted to load dexamethasone into a pH-responsive host-guest nanoparticle, assembled from cyclodextrins and multivalent hydrophilic guest macromolecules. This nanoparticle is able to convert into a hydrogel in the pH conditions of periodontitis, which facilitates drug release and local treatment.

**Figure 5:**
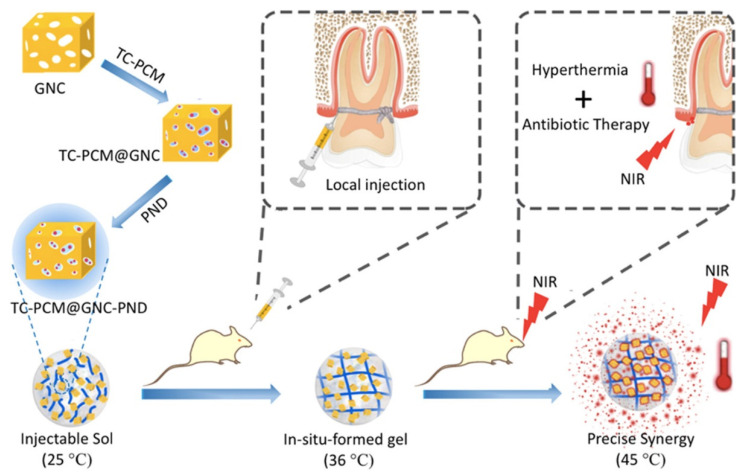
Schematic Illustration of TC-PCM@GNC-PND as a Combined Platform for Hyperthermia and Antibiotics with an Obvious Synergistic Antibacterial Effect. Reprinted with permission from Ref. [61] Copyright 2020 American Chemical Society.

Some hydrogel systems use the photothermal effect to control the release of drugs, combining the antibacterial effect of the photothermal effect with antibacterial drugs to achieve a better antibacterial effect. Zhang et al. [61] fabricated hydrogel platforms for the on-demand release of antibiotics under near-infrared light irradiation. Poly (N-isopropylacrylamide-co-diethylaminoethyl methacrylate) (PND) formed hydrogels in situ at body temperature, which triggered the solid-liquid phase transition of the phase change material through the photothermal effect, resulting in controlled release of tetracycline (Figure 5). Lin et al. [117] similarly used the photothermal effect for periodontal antimicrobial therapy by loading minocycline in GelMA-Au NBPs@SiO_2_ hybrid hydrogel, where near-infrared light irradiation at 808 nm increased the release rate of the drug and controlled bacterial proliferation in periodontal pockets.

In general, hydrogels have been widely used for drug delivery in periodontal disease to achieve antibacterial and periodontal soft and hard tissue regeneration in periodontal pockets. Among them, injectable and temperature-sensitive hydrogels are more frequently chosen in various studies because their physicochemical properties are more suitable for the periodontal pocket microenvironment. To achieve better local targeted drug delivery, specific stimulus-responsive drug release hydrogel systems are also increasingly being used in the treatment of periodontitis.

### 4.4. Maxillofacial Bone Diseases

Maxillofacial bone defect generally emerges after dental removal, implantation, and maxillofacial surgery, which can result in different degrees of damage. The defect resulting from dental extraction and implantation is usually small-scale and faster-healing relatively. However, the defect of mandibular surgery is trickier most of the time.

#### 4.4.1. Mandibular Reconstruction

Mandibular surgery is generally performed in patients with tumor ablation, traumatic infection, and osteonecrosis debridement [118], all of which will carry both great physical sickness and psychological pain to patients. At the moment, the considerable challenges in the clinic include various osteomyelitis, delayed wound healing, mandibular dysfunction, and temporomandibular joint disorder. To overcome these problems, recovering maxillofacial bone defects and promoting bone reconstruction is the key to treatment. Due to its physical structure, great biocompatibility, and controllable bio-degradability [4], the hydrogel has been attempted in mandibular reconstruction and shows enormous potential. To match the complex anatomy and physiology of the mandible, Zhang et al. [119] implemented 3D-printed composite scaffolds combined with dual small molecules, consisting of resveratrol and strontium ranelate. This biological material showed great advantages in promoting angiogenesis and inhibiting osteoclasts. Meanwhile, the application of dual-drug molecules collaboratively promoted mesenchymal stem cell (MSCs) osteogenic differentiation. Aiming at stimulating stem cell differentiation, Lei, L., et al. [60] also developed an injectable hydrogel, which could instantly provide microRNA-222 and aspirin (ASP) at local sites. MicroRNA-222 was proved to be beneficial for the translation of MSCs into neural-like cells by specific signaling. Moreover, ASP played a role in enhancing bone formation as well. The injectable hydrogel incorporated with MSCs and ASP was regarded as promising for innervated bone tissue engineering. What’s more, collagen/nano-hydroxyapatite/alginate (Col/nHA/Alg) hydrogel carrying nerve growth factor (NGF) was developed for increasing new bone formation [120], as well as the hydrogel material impregnated with transforming growth factor-β1 (TGF-β1) and insulin-like growth factor-1 (IGF-1) [121].

BMP-2 proved beneficial in supporting bone formation and was widely applied in various biocompatible scaffolds and matrices [122,123]. A number of studies on mandibular reconstruction fabricated BMP-2 in biological materials. Jung, S.W., et al. [29] developed in situ gelling ALG/HA hydrogels that could release BMP-2 sustainably. The experiments in vitro and in vivo showed that the material has the potential to promote osteogenic differentiation of human bone marrow stem cells (hBMSCs) and the regeneration of mandibular bone. Based on the function of BMP-2, Kim, J., et al. [25] explored the application of electrical stimulation on the bone regeneration of mandibular defect. After electrical stimulation, recombinant human bone morphogenic protein-2 (rhBMP-2) was injected into the scaffold combined with hBMSCs and hydrogels, which proved the combined treatment an effective method. However, not all systems have the function to enhance the effect of BMP-2. The combination of PEG hydrogel and hydroxyapatite/β-tricalcium phosphate (HA/TCP) resulted in the inhibition of BMP-2-induced bone formation compared with the structure in which BMP-2 was loaded with HA/TCP [124]. This study demonstrated that different hydrogels might still exist limitations, which required the scientist’s deeper exploration.

#### 4.4.2. Peri-Implant Diseases

Dental implants have developed rapidly over the years. The correct implantation procedure and postoperative peri-implant osteogenesis are equally significant. Adequate osteogenesis around dental implants inhibits the appearance of peri-implant diseases and makes sure for a healthy post-implant recovery environment [125]. With the number of dental implants increasing, dental practitioners should master the procedures and get a better application of enhancing bone regeneration. Hyaluronic acid hydrogel has been widely used to carry and release BMP-2 to increase peri-implant osteogenesis [26]. An injectable carrying BMP-2 hydrogel system was also prepared, which could sustainably release BMP-2 and showed higher osseointegration levels [37]. Not only the type of materials but also the rate of degradation of the biological material could affect osteogenesis. Meanwhile, one clinic study explored the difference between the application of BMP-2 gel and bisphosphonate gel on the stability of dental implants and marginal bone level, and the consequence showed no significance among the study groups [126]. Akagawa, Y., et al. [127] analyzed the effectiveness of both the fast and slow degradation-type basic fibroblast growth factor (bFGF)-gelatin hydrogel system on peri-implant new bone formation, only to find the latter one reached the highest level of the height of new bone. Beside the BMP-2 and bFGF, the CHX is also applied in the hydrogel to treat peri-implantitis. T. Asbi, et al. [128] reached the conclusion that the application of CHX gel could effectively diminish gingival inflammation during osseointegration in a short-term clinical study. Considering that soft tissue inflammation might lead to peri-implant bone loss in the future, a long-term study should be carried out on the anti-inflammatory effect of CHX gel.

#### 4.4.3. Alveolar Osteitis (Dry Socket)

**Figure 6:**
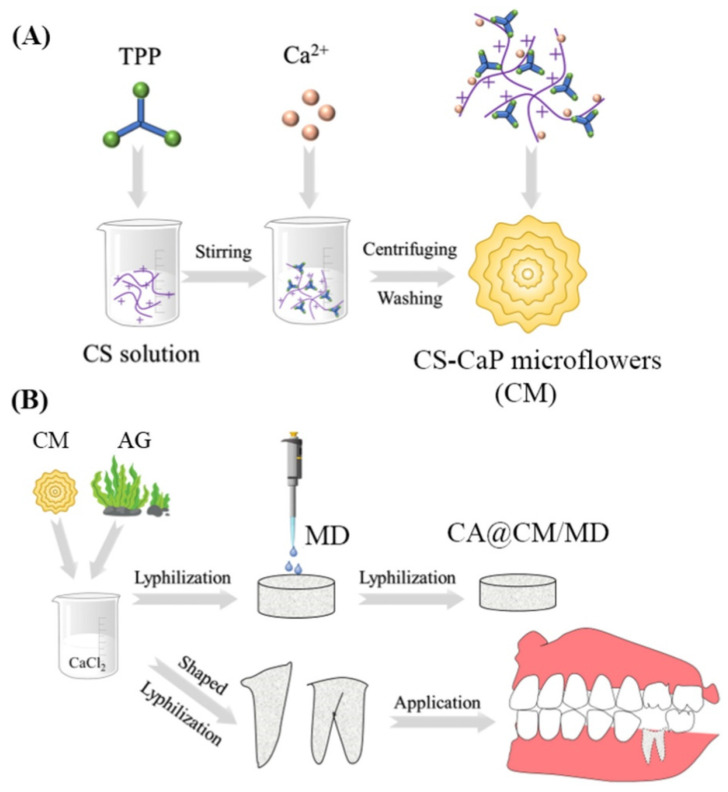
Schematic illustration of the preparation of (**A**) CM and (**B**) CA@CM/MD composite sponges, as well as the application for the prevention of dry sockets after tooth removal. The CM with the porous structure were prepared by combining ionotropic gelation with biomimetic mineralization. After Ca^2+^ crosslinking, lyophilization, and electrostatic interaction, CA@CM/MD composite sponges were fabricated and shaped to the root-like shape for better suitability and applicability. Reprinted with permission from Ref. [132] Copyright 2022 Elsevier.

Alveolar osteitis (dry socket) is one of the most common complications following dental extraction, which has an incidence of approximately 3% for routine treatment, and even more than 30% for impacted mandibular third molars [129]. The etiology of alveolar osteitis is still under exploration, and the mainstream believes that the leading causes are the absence of blood clots and the inflammation of the socket [130]. Hence, the patients are generally requested to take metronidazole orally for 7~14 days after the operation to decrease inflammation. However, long-term antibiotics administration may cause severe side effects, such as vomiting, diarrhea, and constipation. Plenty of materials to prevent alveolar osteitis have been developed. Based on the antioxidant and anti-inflammatory properties of silibinin [131], Xu et al. [42] engineered silibinin into GelMA hydrogels to synthesize the Sil-GelMA. The controlled release of silibinin from the system exhibited an effect of anti-inflammatory and promoted vascularization by inducing the polarization of M2-type macrophage and regulating the secretion of anti-inflammatory factors and VEGF. Calcium alginate (CA) sponges loading with chitosan-CaP microflowers (CM) and metronidazole (MD) (Figure 6) were designed in the shape of the tooth root [132]. It developed the function of anti-bacterial, hemostatic, and osteogenesis, and provided a promising choice for preventing alveolar osteitis.

#### 4.4.4. Osteonecrosis of the Jaw

Osteonecrosis of the jaw (ONJ) has a high incidence in tumor patients, who have to intake high-dose antineoplastic drugs or undergo long-term radiotherapy. According to etiology, ONJ is classified into medication-related ONJ (MRONJ) [133] and osteoradionecrosis (ORN) [134].

MRONJ generally refers to the ONJ resulting from bisphosphonates (BPs) administration which could inhibit the function of osteoclasts and treat benign and malignant bone diseases [135]. It has been proved that BPs have a negative and direct effect on bone cells and vascular tissue, causing necrosis of tissue eventually [136]. Based on the pathogenesis, researchers have come up with plenty of strategies to prevent MRONJ and enhance bone healing. VEGF, which plays a vital part in wound healing, was encapsulated in hydrogels, and then inserted into the maxillary extraction defect [30]. The ultimate outcome showed that the hydrogels combined with VEGF were beneficial to assist bone healing. VEGF demonstrated a pro-angiogenic and immunomodulatory function to prevent the occurrence of MRONJ as expected. Meanwhile, Brierly et al. [137] evaluated the function of another delivery system, which consisted of a poly (ethylene glycol)-heparin hydrogel core and encapsulated molecules including arginyl glycylaspartic acid (RGD) and rhBMP-2. This delivery system was also proved as a potentially useful tool to enhance the formation of bone cells and prevent MRONJ development. Besides VEGF and rhBMP-2, bFGF [138] is also put into an application, which promotes bone formation and inhibits the development of MRONJ.

Long-term exposure to high-dose radiation could lead to terrible adverse effects on the hard and soft tissues of the maxillofacial area. At present, the exact pathogenesis of ORN is uncertain and may be related to reduced wound healing and secondary infection [139]. Consequently, the treatment of ORN is still based mainly on symptomatic therapy, such as the surgical resection of the lesion area and debridement [140]. However, the outcome of conventional therapy is generally unsatisfactory, because the ORN always gets recurrence. Based on this problem, the researchers came up with MSCs and growth factors, which might have the capacity to promote the regeneration of the lesion tissue with delayed wound healing [141]. Combined hydrogels with rat MSCs and BMP-2, to investigate the effect on the osseous healing of ORN, which proved that the application of both MSCs and BMP-2 led to the best effects on enhancing osseous healing [27].

### 4.5. Oral Mucosa Diseases

Oral mucosal diseases are a large group of benign and malignant diseases common in oral lesions and the etiology is complicated and mostly uncertain [142]. Oral mucosal diseases often result in patients’ long-term pain. Hence, clinical doctors should pay more attention to the diagnosis and treatment of oral mucosal diseases.

Due to the complexity and specificity of the oral environment, topical drug administration of oral mucosal diseases has met plenty of limitations and problems. The high moistness and the presence of multiple proteins and mucins prevent robust, stable adhesion of drugs and biomaterials to the buccal mucosa [3]. Therefore, it’s of vital significance to develop a type of biomaterial displaying superior adhesion capability. To promote the properties, Janus patches with a function of wet adhesive were developed to treat the oral ulcer [143]. The researchers were inspired by the components of barnacle and hybrid adhesive mechanism and then combined modified glycine with hydroxyapatite nanoparticles by bridge bonding polymer chains to create a robust adhesive layer. The patch got over the shortcomings of commercial products and achieved analogous instant wet adhesion finally. Meanwhile, Ryu et al. [144] similarly developed chitosan oral patches called Chitoral from the inspiration of mussel adhesion. As well as Janus, Chitoral provided an adhesive layer in oral wet environments. When Chitoral has contact with a mucous layer and saliva, it instantly dissolves into compounds that interact with mucins and form a tough and insoluble adhesion layer between Chitoral and mucous. In the end, Chitoral would get a transformation into adhesive hydrogels under the physical and chemical reactions. Not limited to promoting the property of mucoadhesion, the researchers also endeavor to enhance other functions of biomaterial hydrogels. Ding et al. [145] fabricated a supramolecular hydrogel with mucosal adhesion and anti-oral leukoplakia function under the interaction of isoguanosine-tannic acid. Both isoguanosine and tannic acid have the anti-oral leukoplakia function, and this hydrogel was shown to be a potential platform for inhibiting the deterioration of oral leukoplakia.

**Figure 7:**
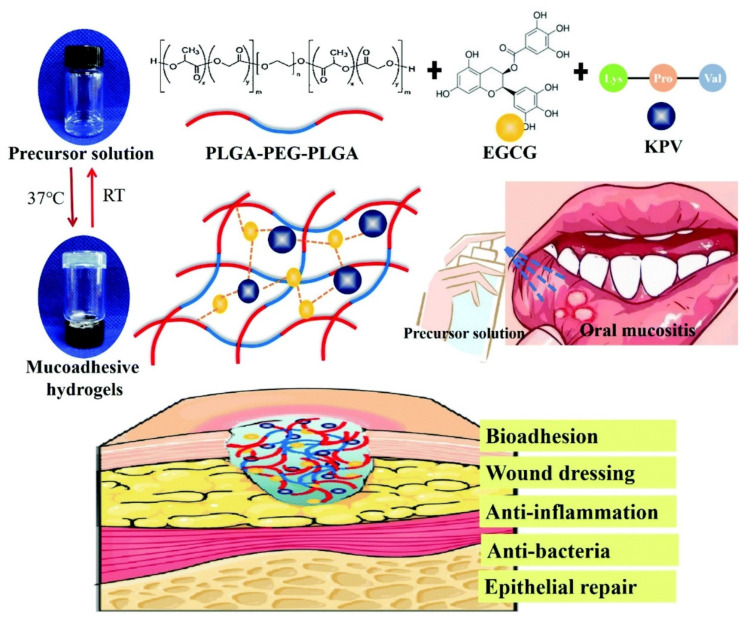
Scheme of mucoadhesive KPV@PPP_E hydrogel for chemotherapy-induced oral mucositis. Reprinted with permission from Ref. [58] Copyright 2022 Royal Society of Chemistry.

As mentioned above, oral mucosal diseases are a group of diseases with complex etiology. One of the most common lesions is an infectious disease caused by fungi or bacteria. However, more and more studies reported drug resistance during the treatment, making it crucial to prepare an advanced anti-fungi/bacteria biomaterial drug at the moment. *Candida* spp. is one of the most prevalent opportunistic pathogens which causes oral candidiasis [146]. Aiming at *Candida* spp., hydrogels fabricated with amino acid substituted histatin-5 (Hst-5) were designed to present the function of anti-fungi and promote wound healing at the same time [147]. Meanwhile, methylcellulose hydrogels loaded with Melissa officinalis oil produced a critical reduction in *C. albicans* [65]. In addition to anti-fungal, Shao et al. exploited temperature-sensitive PLGA-PEG-PLGA as a matrix, adding epigallocatechin-3-gallate (EGCG) as a compound of anti-bacterial and enhancing adhesion, and obtained a promising anti-inflammation application of treating chemotherapy-induced oral mucositis (Figure 7) [58].

### 4.6. Oral Cancer

Currently, oral cancer is the eleventh most common cancer worldwide, most commonly occurring in middle age and older adults. Squamous cell carcinoma is the most common oral malignant tumor, accounting for 90% of oral malignant tumors [148]. In the current treatment of oral cancer, chemotherapeutic drugs such as doxorubicin, cisplatin, and 5-fluorouracil are often used alone or in combination [149,150,151]. The low solubility and low bioavailability of these anticancer drugs are the limiting factors for their use [152]. Therefore, it is necessary to develop different drug delivery systems applied to oral cancer.

In the application of hydrogel systems for oral cancer drug delivery, hydrogels with adhesive or injectable properties are often used to load chemotherapeutic drugs. For instance, Shtenberg et al. [153] mixed alginate and liposomes in different ratios to obtain a hybrid hydrogel with adhesive properties and attempted to use this drug delivery system for sustained release of the anticancer drug doxorubicin (DOX) over a long period of time. Tan et al. [57] mixed a metal-organic framework (MOFs) with thermosensitive hydrogel PLGA-PEG-PLGA into an injectable system to load DOX and celecoxib into the system for local treatment of oral cancer and to achieve stable delivery of two drugs.

**Figure 8:**
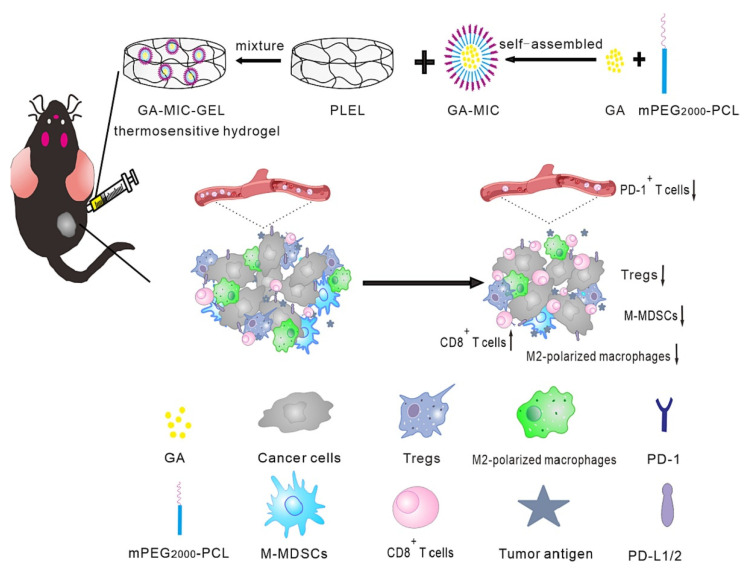
Schematic diagram of the in situ thermosensitive hydrogel containing GA micelles for improving anti-tumor immunity against OSCC. The GA micelle-encapsulated PLEL sol was locally injected into the tumor, formed hydrogel at the body temperature, and continually released GA in situ, thus exerting the chemotherapeutic effect and anti-tumor immune activation. The mice treated with GA-MIC-GEL showed increased cytotoxic T cells as well as reduced immunosuppressive cells at the tumor sites, suggesting the T cell activation and reversal of the tumor immune microenvironment. Besides, the systemic expression of PD-1 in GA-MIC-GEL treated mice also decreased. Reprinted with permission from Ref. [56] Copyright 2022 Elsevier.

Immunotherapy is a recently developed treatment approach that treats disease by strengthening the patient’s immune defenses [154]. Several immune checkpoint blockade treatments have been approved by the FDA, including anti-PD1/PD-L1 and anti-CTLA-4 antibodies [155]. Some studies have explored the local delivery of immunotherapeutic agents in hydrogels. Shi et al. [156] used multidomain peptide (MDP) hydrogels to mimic the natural extracellular matrix and achieved the prolonged release of PD-1 immune-checkpoint inhibitor. Chen et al. [56] loaded gambogic acid (GA) into mPEG200 PCL micelles (MIC), after which GA-MIC was mixed with thermosensitive hydrogels to form injectable hydrogels. The anti-tumor immunity of the tumor-bearing mice was enhanced by down-regulating the expression of PD-1 through the action of GA (Figure 8). At the same time, it’s interesting that both the drug and hydrogel used by Leach et al. [157] had immunotherapeutic effects on tumors. They loaded the immunotherapeutic drug cyclic dinucleotide (CDN) into inducible nitric oxide synthase (iNOS)-inhibited L-nil-MDP hydrogel, which effectively prolonged the median survival of tumor-bearing mice. Besides, it has been found that *Peptostreptococcus* could activate the immune response in oral cancer patients. Zheng et al. [70] used silver nanoparticles combined with an adhesive hydrogel and exogenous bacteria to inhibit the proliferation of bacteria other than *Peptostreptococcus*. This hydrogel system enhanced the anti-tumor immune response by modulating the oral microbiota.

The effect of monotherapy on tumor inhibition is relatively limited, and some studies have combined chemotherapy, radiotherapy, immunotherapy, photothermal therapy, and other therapies to synergistically improve local tumor control and overall survival. Bollareddy et al. [6] combined chemotherapy and immunotherapy. In their study, they applied the chemotherapeutic drug 5-FU and the cyclooxygenase-2 inhibitor Etodolac in combination in transfersomes. These transfersomes prepared from phospholipids and edge activators were loaded in hydroxypropyl methylcellulose (HPMC) hydrogel for local drug delivery in oral cancer treatment. Wu et al. [64] combined chemotherapy and photothermal therapy. They applied near-infrared light (NIR) light-responsive hydrogels to oral cancer treatment by loading DOX into light-responsive mesoporous silica nanoparticles (MSNs), which was loaded into an injectable hydrogel made of methylcellulose together with IR820 as a photothermal agent. The degradation of MSNs was triggered by near-infrared light, which led to the controlled release of DOX. To treat drug-resistant tumors, Alamzadeh et al. [158] combined chemotherapy, radiotherapy, and photothermal therapy by co-loading cisplatin and gold nanoparticles into alginate hydrogel with 532 nm laser and X-ray irradiation. The combination of the three therapies was able to achieve better anti-cancer effects than either monotherapy or the combination of the two therapies.

There have been many attempts at various drug delivery strategies for oral cancer, and currently, it is available to load anti-cancer drugs through different nanoparticles, hydrogels, liposomes, and other carriers for locally targeted drug delivery. At present, most studies are still focused on in vitro or in vivo studies, and there are still few relevant clinical studies. How to apply the existing drug delivery systems in the clinic to achieve lower costs and higher patient survival rates is still a problem that needs to be studied.

### 4.7. Oral Anesthesia

Topical anesthesia is a commonly used method of anesthesia in dentistry to promote a comfortable experience of dental treatment. Effective dental topical anesthesia has many applications in clinical practice, such as relief of postoperative pain, analgesia for oral ulcers, initial periodontal therapy, placement of rubber barrier clip, soft tissue biopsy, etc [159].

At present, the biggest challenge of oral topical anesthesia is how to make the drug reach the target tissue through the oral mucosa epithelium. At present, the main disadvantages of topical anesthetics commonly used in clinical practice are short contact time with the target tissue, limited drug release, and poor anesthetic effect. At the same time, how to safely and effectively prolong the analgesic time of drugs during surgery is also an important issue [160].

Due to their adhesive properties and good biocompatibility, hydrogels have attracted some attention in surface anesthesia [161]. Muniz et al. [69] encapsulated 2.5% lidocaine and prilocaine in poly(ε-caprolactone) nanocapsules and loaded them into CARBOPOL hydrogel. The hydrogel system has satisfactory adhesion ability, and its anesthetic effect is better than that of commercial products. Cubayachi et al. [66] loaded the anesthetics prilocaine hydrochloride and lidocaine hydrochloride into HPMC hydrogels with good adhesive properties, and iontophoresis was used to enhance the penetration and retention of the anesthetics in the mucosa. In addition, by combining microneedles with an adhesive hydrogel, the anesthetic drug can more effectively break through the epithelial barrier for sustained release. Zhang et al. [68] used microneedles and adhesive PAM-PDA-AuNP hydrogels with near-infrared light response in transdermal drug delivery, which reduced pain during anesthesia and could trigger the release of anesthetic drugs by near-infrared light.

Usually, the anesthetic is loaded into the hydrogel to speed up or slow down the release of the drug and to regulate the duration of the drug. Sometimes, rapid drug release is necessary to achieve good anesthesia more quickly. Mihalache et al. [162] prepared interpenetrated/interconnected hydrogels with porous structure and excellent biocompatibility by an amidation reaction between CS and poly[(maleic anhydride)-alt-(vinyl acetate)], which can rapidly release bupivacaine within 15 min, causing a rapid anesthetic effect. On the other hand, by slowing down the drug release and controlling the effective amount of the drug, the anesthetic effect can be effectively prolonged. Ribeiro et al. [163] used xanthan as a hydrogel matrix, employing nanostructured lipid carriers for the delivery of lidocaine-prilocaine for transbuccal pre-anesthesia. The anesthesia time was four times longer compared to commercial products (8 h).

In order to achieve locally controlled delivery of anesthetic and prolong the anesthesia time, temperature-sensitive hydrogels have also been used in some studies. Recently, the thermosensitive injectable hydrogels CS/β-GP were used to controllably release bupivacaine hydrochloride (BH), and graphene oxide (GO) was incorporated into the hydrogel system to alter its chemical and physical properties. Ultimately, the duration of anesthesia was prolonged by 6.5 times in the in vivo study [50]. A hydrogel based on the temperature-sensitive hydrogel, poloxamer 407, has also been used in local anesthesia by incorporating a nanostructured lipid carrier containing bupivacaine into the hydrogel. This hydrogel can transition from liquid to gel at specific concentrations and temperatures and adhere to the mucous to prolong drug retention time. Ultimately, the use of this hydrogel system slowed the penetration of anesthetic agents and extended the duration of analgesia up to three times [160].

### 4.8. Other Oral Diseases

In addition to the oral and maxillofacial diseases mentioned above, a small number of studies have also applied the drug delivery of hydrogels to areas that have received less attention, such as orthodontic tooth movement, temporomandibular joint disorders, Sjögren’s syndrome, etc., with equally excellent therapeutic results.

Orthodontic tooth movement is a complex biological process that involves remodeling the alveolar bone and periodontal ligaments in response to orthodontic force stimulation. How to improve orthodontic tooth movement by regulating the process of bone remodeling is an important issue. Some studies have made attempts through hydrogel drug delivery systems to solve this issue. Xing et al. [164] used a nanofibrous self-assembled peptide hydrogel for the sustained and controlled release of RANKL protein to induce local osteoclastogenesis and facilitate the bone remodeling process of orthodontic tooth movement. Lu et al. [59] used PEG-PCL-PEG temperature-sensitive hydrogel for the controlled release of parathyroid hormone (PTH) or parathyroid hormone-related protein (PTHrP), which was able to enhance orthodontic tooth movement in rats by modulating bone remodeling.

Temporomandibular joint disorders (TMD) are a group of diseases including masticatory muscle disorders, structural disorders of the temporomandibular relationship, inflammatory diseases, and osteoarthropathies [165]. Intra-articular injection of hyaluronic acid is often applied in the treatment of TMD [166]. Chitosan-based thermosensitive hydrogels can be used to control the release of hyaluronic acid within the TMJ, slowing the clearance of hyaluronic acid from the injected portion and thus reducing the number of injections [52].

Sjögren’s syndrome is a chronic autoimmune disease with the involvement of the exocrine glands. Involvement of the salivary and lacrimal glands usually causes dryness of the mouth and eyes [167]. Pilocarpine hydrochloride as the drug of choice for the treatment of dry mouth or dry eyes caused by Sjögren’s syndrome [168] has also been attempted in studies using hydrogels for drug delivery. Gelatin can be used to load the highly soluble drug pilocarpine hydrochloride, which was able to effectively reduce the rate of drug release and had a significantly prolonged effect on tear formation [169].

The sustained antimicrobial effect in the complex bacterial or fungal environment of the oral cavity is significant. In the oral environment, sustained release of antimicrobial drugs is essential for effective bacterial inhibition. Ribeiro et al. [170] loaded chlorhexidine in nanotubes and used cytocompatible GelMA hydrogel to provide sustained release of CHX. In another study, the releasing period of cetylpyridinium chloride (CPC) can be extended by loading CPC into HEMA/TMPT hydrogels to achieve sustained antibacterial activity [73]. Loading ciprofloxacin with fibrin hydrogel allows the gradual release of ciprofloxacin over 168 h. In contrast, alginate hydrogel released all ciprofloxacin within 1 h [171].

To date, for diseases that have received less attention, hydrogels have been used as drug delivery vehicles in a small number of studies, and the application of hydrogels in them deserves more in-depth study.

## 5. Perspective and Conclusions

It is necessary to acknowledge that this review can hardly avoid bias and errors to some extent, as there is no uniform evaluation of the scientific validity of the design, methodology, and results of the original literature such as a systematic review. However, it is still informative to learn about the application and research progress of hydrogels in maxillofacial and oral drug delivery. Compared with other materials such as nanoparticles, nanofibers, and thin films, many hydrogels have biocompatibility and unique stimulus-responsive properties that make them suitable as carriers or platforms for transporting drugs, cells, and others to target locations, which have unique advantages in local therapies and are therefore well suited for topical applications in the oral environment. This paper describes the application and research progress of hydrogels in maxillofacial and oral drug delivery. Different types of hydrogels have a wide range of applications in oral soft and hard tissue regeneration, antibacterial, and local drug targeting delivery due to their injectability, temperature sensitivity, pH sensitivity, biodegradability, and other properties. Many hydrogels do not have the function of repairing tissue defects themselves, but can be used as carriers for different drugs, growth factors, and even stem cells to achieve higher concentrations maintained over a long period of time. Some hydrogel systems are able to respond to stimuli such as temperature, pH, and light irradiation, which can modulate the drug release from the hydrogel on demand.

In the future, how to obtain hydrogel systems with greater biocompatibility through smaller cost and simpler synthesis methods to achieve superior sustained local drug delivery efficacy are still important issues for researchers to focus on. There are many hydrogel drug delivery systems made with different materials and synthesis methods for complex oral and maxillofacial environments, but the high costs, complex synthesis steps, and toxic biodegradation by-products may be the key challenges affecting their further clinical applications. Meanwhile, in vivo studies and animal studies are relatively scarce, and the exploration of relevant hydrogels for clinical applications is even rarer. The stability of topical drug delivery and the biocompatibility of hydrogels need to be further investigated, and their use in the clinic requires more rigorous testing. It is expected that more clinical studies will be conducted in the future to verify the therapeutic effects of hydrogels as drug release platforms in different oral diseases, especially in the treatment of periodontal diseases, which currently have a relatively large number of clinical studies.

## Figures and Tables

**Table 1:** The characteristics of the studies using hydrogels with special properties in this review.

Classification	Hydrogels	Crosslinking Method	Drug-Loading Ways	Active Ingredients	Application	Characteristic	References
Injectable	Fibrin	Physical crosslinking	Poly (d,l) Lactic Acid nanoparticles	Clindamycin	Regenerative endodontics and antibacterial	Excellent cytocompatibility; physiological degradation kinetics; non-toxicity of degradation products; replacement with cell-derived ECM within a few days.	[23]
	Hyaluronic acid (HA)	Chemical crosslinking	/	rhBMP-2; rMSCs and BMP-2; platelet lysate, chemotactic and pro-angiogenic growth factors (PDGF and VEGF)	Regenerative endodontics; Mandibular reconstruction; Peri-implant osteogenesis	Controlled release of drugs; supportive matrix for cell culture, recruitment, and revascularization induction.	[24,25,26,27]
	Alginate	Chemical crosslinking	/	Vancomycin/deferoxamine/dexamethasone (Van/DFO/Dex)	Maxillofacial bone regeneration	Locally sustained release property; prominent biological functions.	[28]
	Alginate (ALG)/hyaluronic acid (HA)	Chemical crosslinking	/	BMP-2	Mandibular reconstruction	In situ gelling hydrogel with a controllable gelation rate using CaSO_4_ as a crosslinking agent and Na_2_HPO_4_ as a crosslinking retardation agent.	[29]
	Gelatin-hyaluronic acid hydrogel	Chemical crosslinking	/	Vascular endothelial growth factor (VEGF)	medication-related ONJ (MRONJ)	Assists bone healing and prevents MRONJ via a pro-angiogenic and immunomodulatory mechanism.	[30]
	−CHO inaldehyde-modified hyaluronic acid (HA-CHO) and −NH_2_ in glycol chitosan (GC), Fe^3+^	Chemical crosslinking	/	Ginsenoside Rg1 and amelogenin	Periodontal disease	Injectable and self-healing hydrogels with double-dynamic bond tunable mechanical, gel-sol transition, and drug delivery properties.	[31]
	Oxidized alginate/carboxymethyl chitosan	Chemical crosslinking	/	A dental epithelial cell line, HAT-7	Dental Enamel Regeneration	The Self-Crosslinkable hydrogels could be used as an injectable cell carrier for dental enamel tissue engineering applications.	[32]
	Carboxymethyl-chitosan and a diglycidyl ether	Chemical Crosslinking	/	Calcium phosphate nanoparticles	Pulp capping	These composites have moduli up to 3 MPa, and support the culture of dental pulp stem cells for more than 3 weeks.	[33]
	The chitosan hydrogel as well as blends of polyvinyl pyrrolidone (PVP), polyvinyl alcohol (PVA) and PEG	Chemical crosslinking	/	Insulin	Periodontal bone regeneration	A linear hydrogel that is injectable into periodontal pockets, and is able to carry a small insulin load through physical bonds and provide sustained release.	[34]
	Oxidized dextran (OD) and phenylboronic acid-functionalized poly (ethylene imine) (PBA-PEI)	Chemical crosslinking	/	Doxycycline and metformin	Periodontal antibacterial, anti-inflammatory and bone regeneration	Simultaneously improving drug loading efficiency (doxycycline and metformin) through B−N coordination and achieve ROS triggered drug release locally.	[35]
	poly(lactide-co-glycolide) (PLGA) and N-methylpyrrolidone (NMP)	Chemical crosslinking	/	Minocycline (MCL)	Periodontal antibacterial	Exhibited the characteristic of Newton fluid with acceptable syringeability. Drug release could last for more than 48 h with an acceptable “burst release”.	[36]
	poly(phosphazene)	Physical crosslinking	/	BMP-2	Peri-implant osteogenesis	Vertical bone regeneration and higher osseointegration levels.	[37]
	polyisocyanopeptide (PIC)	Physical crosslinking	PLGA microspheres	Doxycycline and lipoxin	Periodontal anti-inflammatory and antibacterial	Appropriate injectability; long-term structural stability; the release profiles of drugs could be manipulated by adjusting the loaded mass ratio of acid- and ester- terminated PLGA microspheres in the PIC gels.	[2,38]
	Gelatin methacrylate (GelMA)	Chemical crosslinking (Photocrosslinking)	(Au NBPs@SiO (2)) or none	Chlorhexidine (CHX); pro-angiogenic growth factors (GFs); BMP-mimetic peptide; minocycline; silibinin	Regenerative endodontics; Periodontal antibacterial; Prevention of dry sockets	Cytocompatible; biodegradable; provides sustained release of drugs.	[39,40,41,42]
	MA-HA/di-thiol PEG	Chemical crosslinking (Photocrosslinking)	/	A novel, small-molecule noncompetitive adenosine triphosphate (ATP) drug: NP928, belongs to the thiadiazolidinone (TDZD) family	Dentin Regeneration	Biodegradable; gelling in situ upon dental blue light exposure.	[43]
	Polyethylene glycol-maleate-citrate (PEGMC)	Chemical crosslinking (Photocrosslinking)	/	Calcium hydroxide	Direct pulp capping	The light-curing time for hydrogel is comparable to composite resin. Controlled Ca^2+^ release was obtained.	[44]
	Methacrylated-poly-γ-glutamic acid (MPGA) polymer	Chemical crosslinking (Photocrosslinking)	Methacrylated-poly-γ-glutamic acid nanoparticles (PGA-MNP)	Metronidazole and CHX	Periodontal antibacterial	It is a pH-sensitive drug delivery system which used blue-light photopolymerization for preparation.	[45]
Injectable and thermosensitive	Pluronic F127 (PF127)	Chemical crosslinking (thermal crosslinking)	/	Iloprost; Simvastatin; glycogen synthase kinase 3 beta inhibitor (BIO); Metronidazole	Regenerative endodontics; Periodontal anti-inflammatory and osteogenic	Controlled drug release; could adhere to hard tissue and gradually release.	[46,47,48,49]
	chitosan (CS)/β-glycerophosphate (GP)	Physical crosslinking	Graphene oxide (GO) nanosheets or none	Bupivacaine hydrochloride (BH); VEGF; hyaluronic acid; bone morphogenetic protein-7 (BMP-7) and ornidazole (ORN); Naringin; aspirin and erythropoietin (EPO); quercetin	Topical anesthesia; Pulp capping; Temporomandibular disorders; Periodontal anti-inflammatory and tissue regeneration	Prolonged drug release time; a stable and sustained drug release system.	[50,51,52,53,54]
	Chitosan/gelatin/glycerol phosphate	Chemical crosslinking	/	BMP-6	Periodontal tissue regeneration	Provide a 3D environment for transplanted stem cells and to enhance stem cell delivery and engraftment.	[55]
	poly(D, L-lactide)-poly(ethylene glycol)-poly(D, L-lactide) (PLEL)	Chemical crosslinking	mPEG2000-PCL micelles	Gambogic acid (GA)	Oral cancer	The thermosensitive GA-MIC-GEL with sensitive sol-gel transition characteristics could form hydrogel at 37 °C within 24 s, facilitating the local delivery and sustained GA release.	[56]
	poly(D,L-lactide-coglycolide)-poly(ethy-lene glycol)-poly(D,L-lactide-coglycolide) triblock copolymers (PLGA-PEG-PLGA)	Chemical crosslinking	/	Doxorubicin (DOX) and celecoxib	Oral cancer	pH-responsiveness; biocompatibility; simultaneously release hydrophobic and hydrophilic drugs at the oral tumor site.	[57]
	PLGA-PEG-PLGA (PPP)/epigallocatechin-3-gallate (EGCG)	Chemical crosslinking	/	Tripeptide KPV	Oral mucosal disease	In situ mucoadhesive; anti-inflammatory, antibacterial and repairing effect on chemotherapy-induced oral mucositis.	[58]
	poly(ethylene glycol)-poly(ε- caprolactone)-poly(ethylene glycol) (PEG-PCL-PEG, PECE)	Chemical crosslinking	/	Parathyroid hormone (PTH) or parathyroid hormone-related protein (PTHrP)	Orthodontic tooth movement	Aqueous solution of PECE copolymers changed from the “sol” phase to the “gel” phase with the increase in temperature.	[59]
	poly(ethylene glycol)-6-poly(lactic-co-glycolic acid)-6-po(y(N-isopropy!acrylamide) (PEG-PLGA-PNIPAM) hydrogel	Chemical crosslinking	Mesoporous silica nanoparticle (MSN)-embedded core-shell structure	MicroRNA-222 and ASP	Mandibular reconstruction	Injectable colloidal hydrogel with mesoporous silica nanoparticles for sustained co-release of microRNA-222 and aspirin	[60]
	poly(N-isopropylacrylamide-co-diethylaminoethyl methacrylate) (PND)	Chemical crosslinking	Gold nanocages (GNC)	Tetracycline(TC)	Periodontal antibacterial	Near infrared light (NIR) light controlling drug release through the dual thermosensitive interaction of liquid-solid transition of PCM and coil-granule transition of PND.	[61]
	Self-assembling peptides (SAP) hydrogel (P11-4 and P11-28/29)	Physical crosslinking	/	Tetracycline, ciprofloxacin, and doxycycline	Periodontal tissue regeneration and antibacterial	Biocompatibility; cargo-loading capacity; tunable physicochemical and mechanical properties.	[62]
	Polyethylene glycol diacrylate (PEG-DA) based scaffolds, dithiothreitol (DTT), and a novel designed functional peptide module (FPM)	Chemical crosslinking	/	Stromal cell derived factor-1 (SDF-1)	Periodontal tissue regeneration and antibacterial	PEGPD@SDF-1 hydrogel exhibited preferable biocompatibility and could promote the proliferation, migration, osteogenic differentiation of periodontal ligament stem cells (PDLSCs) and inhibit the growth of *Porphyromonas gingivalis*.	[63]
Adhesive	Methylcellulose	Physical crosslinking	Mesoporous silica nanoparticles (MSNs) or none	DOX; Melissa officinalis oil	Oral cancer; Oral mucosal disease	Biocompatibility; controllable mechanical performance; thermosensitive and injectable characteristics.	[64,65]
	Hydroxypropyl methylcellulose (HPMC)	Chemical crosslinking	Transfersomes or none	Prilocaine hydrochloride and lidocaine hydrochloride; 5-Fluorouracil and Etodolac; polyaspartic acid-stabilized amorphous calcium phosphate (PAsp-ACP) nanoparticles	Topical anesthesia; Oral cancer; Biomimetic mineralization	HPMC can be desiccated to form a dry film. In a moist environment, this film gradually changes into a gel.	[6,66,67]
	PAM-PDA	Physical crosslinking	AuNPs	Medical anesthetic	Topical anesthesia	This hydrogel with microneedle resulted in reduced pain, higher anesthetic accuracy and faster recovery.	[68]
	Carbopol	Physical crosslinking	Poly(ε-caprolactone) nanocapsules	Lidocaine and prilocaine	Topical anesthesia	Non-Newtonian pseudoplastic flows; satisfactory mucoadhesive strength; non-cytotoxicity; slow permeation across oral mucosa.	[69]
	Polyaldehyde dextran and chitosan	Chemical crosslinking	/	Silver nanoparticles	Oral cancer	Antitumor responses were enhanced by the subcutaneous delivery of an adhesive hydrogel incorporating silver nanoparticles (which inhibited the growth of bacteria competing with *Peptostreptococcus*).	[70]
	Acrylic acid/polyethylene glycol	Chemical crosslinking	/	Silver nanoparticles and propranolol HCl	Antibacterial	The nanocomposites show a promising self-disinfection property and mucoadhesive strength.	[71]
Expansive	N-vinylpyrrolidone(NVP), 2-hydroxyethyl methacrylate (HEMA), and glycerolmonomethacrylate (GMMA) monomers with methacrylicacid (MA).	Chemical crosslinking	/	Benzocaine	Anesthesia of tissue expanders	Tissue expanders based on the controlled rate expansive hydrogels. Most of the drug (90%) was released within 48 h.	[72]
Nondegradable	2-hydroxyethyl methacrylate (HEMA) and trimethylolpropane trimethacrylate (TMPT)	Chemical crosslinking	/	Cetylpyridinium chloride (CPC)	Antibacterial	Applying a non-biodegradable hydrogel to resin-based materials as a reservoir for water-soluble antimicrobials	[73]

## Data Availability

Not applicable.
